# PP88 Breaking Boundaries In Health Technology Assessment: Quantifying Non-Traditional Value Of Medical Technology Through ExpertLink Remote Connectivity Solutions

**DOI:** 10.1017/S0266462324002563

**Published:** 2025-01-07

**Authors:** Callix Wong, Dinesh Patel, Michael Cheng, Tze Wei Khor, Devindran Manoharan, Selvalingam Sothilingam, Hui Meng Tan, Georgia Swan

## Abstract

**Introduction:**

A diverse range of services often supplements procedures that involve medical technologies and adds value along patient care pathways. However, these novel elements of value are often not captured in traditional assessment frameworks. ExpertLink is one such example. ExpertLink uses digital solutions to connect clinical experts worldwide, enabling remote training and collaboration, while maintaining the highest standard of patient care.

**Methods:**

Rezum™ is a minimally invasive therapy for patients with symptomatic benign prostatic hyperplasia (BPH). It is a quick day procedure with proven safety, effectiveness, and durability in clinical outcomes. Leveraging ExpertLink, an expert in Sydney, Australia, remotely proctored 11 Rezum™ procedures in Malaysia in November 2022, supporting five urologists in five hospitals across five states within five hours. Efficient and straightforward procedures such as Rezum™ are well suited to remote proctorship. Through this case study, we quantify the sustainability, equity, and access benefits, illustrating the additional value ExpertLink brings across the healthcare continuum and beyond.

**Results:**

For a proctor traveling from Australia to Malaysia, over 6,500 kilometers and 17 hours travel time is saved, equating to an estimated 1,700-kilogram reduction in CO₂ emissions. Without ExpertLink, a proctor may be away from practice for up to a week. ExpertLink allows for continuity of practice, including consultations and procedures, during this time. For five doctors traveling from Malaysia to Australia for training, an estimated 7,400-kilogram reduction in CO₂ emissions and approximately 85 hours travel time is saved. ExpertLink provided 11 geographically dispersed patients with timely access to treatment and expedited the physician learning curve.

**Conclusions:**

This case study illustrates the value for just one technology on one day. ExpertLink embodies novel elements of value that are not captured in traditional value assessment frameworks. Collaborative effort between stakeholders is needed to broaden the view of value in healthcare, incorporate additional elements of value in existing assessment frameworks, and appropriately recognize this often-uncounted value in decision-making.

